# EZH2 as a Potential Target for NAFLD Therapy

**DOI:** 10.3390/ijms21228617

**Published:** 2020-11-16

**Authors:** Hyun Jung Lim, Mirang Kim

**Affiliations:** 1 Personalized Genomic Medicine Research Center, Korea Research Institute of Bioscience and Biotechnology (KRIBB), Daejeon 34141, Korea; hjlim@kribb.re.kr; 2 Department of Functional Genomics, University of Science and Technology (UST), Daejeon 34113, Korea

**Keywords:** epigenetics, EZH2, NAFLD, NASH, liver fibrosis

## Abstract

Non-alcoholic fatty liver disease (NAFLD) is a complex disease that is affected by genetic predisposition and epigenetic modification. Deregulation of epigenetic pathways is now recognized as a frequent event in NAFLD, and understanding the mechanistic roles of these epigenetic factors may lead to new strategies for NAFLD treatment. Enhancer of zeste homolog 2 (EZH2) catalyzes methylation on Lys 27 of histone H3, which leads to chromatin compaction and gene silencing. EZH2 regulates embryonic development and cell lineage determination and is related to many human diseases. Recent studies show that EZH2 has critical roles in liver development, homeostasis, and regeneration. Moreover, aberrant activation of EZH2 promotes NAFLD progression. Several EZH2 inhibitors have been developed and studied both in vitro and in clinical trials. In this review, we summarize our current understanding of the role of EZH2 in NAFLD and highlight its potential as a novel therapeutic target for NAFLD treatment.

## 1. Introduction

Non-alcoholic fatty liver disease (NAFLD) is the most common liver disease worldwide [1]. The worldwide prevalence of NAFLD is ~25%, and its incidence has increased dramatically with the global epidemic of obesity [2]. NAFLD pathogenesis has been described extensively [3]. Initial theories for the pathogenesis of NAFLD were based on a two-hit hypothesis. According to this hypothesis, hepatic accumulation of lipid (the first hit) increases the susceptibility of the liver to further insults mediated by second hits. These second hits, such as inflammatory cytokines, mitochondrial dysfunction, and oxidative stress, lead to steatohepatitis and fibrosis [4,5]. However, human NAFLD is more complicated with the involvement of multiple factors, and now a multiple-hit hypothesis has been accepted to explain the progression of NAFLD [6]. 

In 1980, the term NAFLD was first coined to describe fatty liver disease arising in the absence of significant alcohol intake [7]. However, the heterogeneity of NAFLD patients suggests the need for a more accurate term that can reflect pathogenesis and can help with patient classification for therapy. Recently, metabolic (dysfunction) associated fatty liver disease (MAFLD) was suggested as a more appropriate overarching term for NAFLD [8].

NAFLD has several stages in its progression that can lead to non-alcoholic fatty liver (NAFL), non-alcoholic steatohepatitis (NASH), cirrhosis, and even hepatocellular carcinoma (HCC) [9]. Whereas NAFL is a simple steatosis, NASH is characterized by cell injury, inflammation, and hepatocyte ballooning that may further progress to fibrosis, cirrhosis, and HCC. Although patients with NAFL have a similar life expectancy to that of the general population, those with NASH have an impaired survival, due primarily to cardiovascular and liver-related causes [10]. High-fat, high-sugar, and hypercaloric diets increase the risk of hepatic steatosis [11]. By contrast, weight loss achieved by caloric restriction reduces hepatic inflammation and fibrosis [12]. Current NAFLD management includes diet and lifestyle changes for weight loss, management of metabolic risk factors, and pharmacological treatment [13]. The purpose of these treatments is to prevent NAFLD-associated complications [14]. Currently, there are no FDA-approved therapies for NAFLD itself, highlighting the urgent need to find appropriate targets for NAFLD therapy [15]. Treatment of NAFLD is difficult because the progression of NAFLD is a multi-factorial process [16]. Recently, our understanding of these pathways has increased substantially, along with our ability to antagonize them, and the development of new drugs for NAFLD patients is actively underway. 

Diet, lifestyle, and environmental and genetic factors can lead to insulin resistance (IR), obesity, and gut microbiome changes. IR is one of the main factors in the development of steatosis and NASH. IR increases hepatic de novo lipogenesis and increases the flux of fatty acids to the liver, with impaired inhibition of adipose tissue lipolysis [17]. The synthesis and accumulation of triglyceride and toxic levels of lipid metabolites lead to hepatic inflammation, which results from mitochondrial dysfunction through oxidative stress and endoplasmic reticulum stress through activation of the unfolded protein response [18]. Therefore, genetic or epigenetic factors affect hepatocyte fat content, enzymatic processes, and the liver inflammatory environment, thus leading to inflammation and fibrosis [6]. 

Epigenetic modifications are heritable (and yet also reversible) modifications of the genome that do not involve a change in the DNA sequence [19]. Various enzymes and other proteins are involved in epigenetic modifications [20]. Aberrant epigenetic changes can lead to inappropriate gene expression and can promote many chronic diseases including NAFLD. As epigenetic modifications are reversible, chromatin-modifying enzymes have emerged as promising targets for disease therapy. Recently, various drugs targeting epigenetic regulators have been developed and used in clinical trials as well as in preclinical studies [21]. Enhancer of zeste homolog 2 (EZH2) is a histone lysine methyltransferase that is involved in many human diseases, most of which are types of cancer [22]. Recent epigenetic studies revealed that EZH2 has important roles in liver homeostasis and NAFLD progression. In this review, we highlight EZH2 as a potential target for NAFLD therapy. 

## 2. Epigenetic Features of NAFLD

### 2.1. DNA Methylation in NAFLD 

NAFLD is a complicated disease associated with genetic and epigenetic factors [23]. Genetic variation in *PNPLA3* is one of the common genetic risk factors of NAFLD [24]. PNPLA3 is a lipid droplet-associated protein that has hydrolase activity toward triglycerides and retinyl esters. The PNPLA3 I148M variant (rs738409 C>G) is associated with hepatic triglyceride accumulation, inflammation, and fibrosis. Multiple genome-wide association studies and epigenetic studies have been conducted during the past decade, which have enhanced our understanding of the genetic and epigenetic factors contributing to NAFLD progression. The focus of this review is on these epigenetic mechanisms.

Environmental factors such as diet, lifestyle, and the gut microbiome affect NAFLD progression by inducing aberrant epigenetic changes. Epigenetic alterations interact with genetic risk factors to determine an individual’s overall risk for NAFLD [25]. Epigenetic changes are reversible and heritable modifications that do not involve changes in the DNA sequence. The main epigenetic mechanisms involve DNA methylation, histone modifications, and non-coding RNAs. DNA methylation is the covalent addition of a methyl group to cytosine, resulting in 5-methylcytosine. DNA methyltransferases (DNMT1, DNMT3A, and DNMT3B) catalyze this reaction. DNA methylation is a relatively stable epigenetic mechanism that can regulate gene expression patterns to establish cell identity [26]. Methylation of CpG dinucleotides in promoter regions typically inhibits transcription.

Translational studies on human livers have indicated that NAFLD is associated with abnormal DNA methylation [23,27]. Advanced NAFLD is associated with decreased methylation of tissue repair genes and increased methylation of metabolic pathway genes [27]. The NAFLD liver shows hypermethylation and down-regulation of genes involved in mitochondrial function, lipid metabolism, and oxidoreductase activity, whereas tumorigenesis-related genes are hypomethylated and up-regulated [28]. Interestingly, Ahrens et al. [29] demonstrated that NAFLD-specific methylation patterns were partially reversed after massive weight loss induced by bariatric surgery. Moreover, epigenetic states can vary from person to person depending on genetic background. For example, methylation of the *PNPLA3* regulatory region is affected by the *PNPLA3* rs738409 genotype [30], and methylation of *FAD2* is associated with the *FAD2* variant rs174616 [31]. To sum up, reprogramming of DNA methylation occurs during NAFLD progression and may be affected by an individual’s genotype as well as by environmental factors. 

### 2.2. Histone Modifications in NAFLD

Modification of the amino-terminal tail of histones, such as histone acetylation and histone methylation, can result in changes in the chromatin structure and gene expression. Accumulating evidence demonstrates that excess nutrition and metabolic pathways can trigger histone modification as well as DNA methylation through deregulation of epigenetic regulatory enzymes [32]. By using co-substrates supplied by the diet or generated through cell metabolism, epigenetic regulatory enzymes provide a potential association between nutrition, metabolism, and transcriptional regulation [32,33]. 

Histone acetylation is a highly reversible epigenetic modification and is usually associated with open chromatin and transcriptional activation. Acetylation of a histone increases its negative charge, thereby reducing the strength of its interaction with negatively charged DNA. Histone acetyltransferases (HATs) are “writers” of histone acetylation, whereas histone deacetylases (HDACs) are “erasers” that remove the acetyl group from an acetylated lysine residue [34]. 

HDACs are increased in chronic liver disease, and HDAC inhibitors have been reported to suppress hepatic stellate cell (HSC) activation and lead to the suppression of liver fibrosis [35,36]. Among HDACs, HDAC8 has a key role in NAFLD-associated HCC [37]. HDAC8 is directly up-regulated in dietary obesity models of NASH and HCC [37]. HDAC8 interacts with EZH2 to repress Wnt antagonists *AXIN2*, *NKD1*, *PPP2R2B*, and *PRICKLE1*, leading to β-catenin activation and consequent cell proliferation via histone H4 deacetylation and trimethylation on Lys 27 of histone H3 (H3K27me3) [37]. 

The bromodomain and extra-terminal domain (BET) family proteins (BRD2, BRD3, BRD4, and BRDT) are “readers” of acetylated histone and non-histone proteins [38]. The BET family proteins modulate the promoter and enhancer activity of pro-inflammatory genes [39]. Small-molecule BET inhibitors have been developed in clinical and preclinical studies [40], and BET inhibition reverses fibrotic progression and prevents liver fibrosis in the NASH mouse models [41]. 

Histone methylation can occur at various sites in histone proteins, mainly on lysine and arginine residues. Histone methylation is controlled by multiple positive and negative modulators, which can activate or repress transcription [42]. Methylation of H3K4, H3K36, and H3K79 is often associated with transcriptional activation. By contrast, methylation of H3K9, H3K27, and H4K20 is associated with transcriptional repression. In addition, histone methylation can also influence DNA methylation and vice versa [32].

Histone methylation has an important role in NAFLD. The expression of specific histone lysine methyltransferases (KMTs) and demethylases (KDMs) is altered during NAFLD development [43]. Bricambert et al. [44] demonstrated that a histone demethylase, plant homeodomain finger 2 (Phf2) acts as a molecular checkpoint to prevent NAFLD progression by specifically erasing H3K9me2 (dimethylation on Lys 9 of H3) marks on the promoter of carbohydrate response element binding protein (ChREBP)-regulated genes during obesity. Moreover, histone methyltransferases such as MLL1 [45] and ASH1 [46] are also associated with liver fibrosis. In particular, there is growing evidence that EZH2 has a driving role in NAFLD [47]. 

## 3. Role of EZH2 in the Liver

### 3.1. Polycomb Group (PcG) Protein EZH2

**Figure 1:**
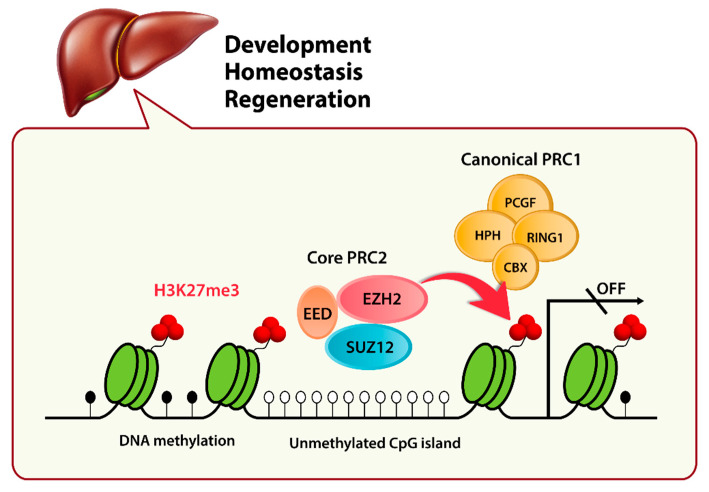
Role of EZH2 in the liver. The core polycomb repressive complex 2 (PRC2) contains suppressor of zeste 12 (SUZ12), embryonic ectoderm development (EED), and the enzymatic catalytic subunit EZH2. PRC2 is recruited to unmethylated CpG islands in repressed genes. EZH2 catalyzes trimethylation on Lys 27 of histone H3 (H3K27me3). H3K27me3 can act as a docking site for the chromobox domain (CBX) protein subunits of PRC1, which leads to chromatin compaction and gene silencing. Canonical PRC1 components include the ubiquitin ligase ring finger protein RING1 and other subunits, such as the human polyhomeotic homolog (HPH) and the polycomb group ring finger protein (PCGF). EZH2 has critical roles in liver development, homeostasis, and regeneration.

PcG proteins are evolutionarily conserved epigenetic regulators that are involved in transcriptional silencing and maintaining cell identity [48]. PcG proteins are grouped into two complexes, polycomb repressive complex 1 (PRC1) and PRC2. PRC2 binds chromatin and catalyzes H3K27me3. H3K27me3 is then recognized by PRC1, which leads to chromatin compaction and gene silencing [49] (Figure 1). The repressive histone mark H3K27me3 is frequently found with the active histone mark H3K4me3 (trimethylation on Lys 4 of histone H3) in the promoters of genes involved in developmental regulation. These regions are referred to as “bivalent domains” and have been suggested to silence developmental genes while keeping them poised for activation during cellular differentiation [50]. The human PRC2 contains suppressor of zeste 12 (SUZ12), embryonic ectoderm development (EED), and the enzymatic catalytic subunit EZH1 or EZH2 [51]. EZH2 is highly expressed in proliferating cells, whereas EZH1 is more abundant in non-dividing adult organs (such as kidney, brain, and skeletal muscle tissue) and has lower enzymatic activity [52]. 

As a catalytic subunit of PRC2, EZH2 is mainly known as a histone lysine methyltransferase, but it can also methylate non-histone proteins such as transcription factors involved in cell adhesion and migration [53], cell growth and apoptosis [54], actin polymerization [55], or T-cell development [56]. In addition, EZH2 can function as a transcriptional activator in a polycomb-independent manner [57]. EZH2 has important roles in embryonic development and cell lineage determination [58] and is a key player in the cell cycle [59], autophagy, and apoptosis [60]. Therefore, EZH2 is related to many human diseases, including NAFLD, as well as cancer [22]. 

### 3.2. EZH2 in Liver Cell Differentiation and Liver Homeostasis

The liver has a tremendous ability to regenerate after partial resection and chemical injury [61]. The mechanism of liver regeneration depends on the proliferation of existing hepatocytes, homing of bone marrow cells, and the proliferation and differentiation of hepatic progenitor cells [62]. Hepatic progenitor cells actively proliferate in the fetal mouse liver and are the main source of parenchymal cells [63]. EZH2 has a key role in the proliferation and differentiation of hepatic progenitor cells [64]. EZH2 is highly expressed in hepatic progenitor cells and controls their expansion [65]. Deletion of the SET domain of EZH2 in fetal mouse liver results in a significant reduction in the total liver size and in the inhibition of hepatocyte differentiation [65]. Moreover, double knockout of EZH1 and EZH2 in mouse hepatocytes causes a decreased level of H3K27me3 in the promoter of genes associated with hepatocyte homeostasis and regeneration [66]. Grindheim et al. [67] suggested that EZH2 controls the timing of postnatal hepatocyte maturation. Liver-specific disruption of EZH1 and EZH2 leads to immature differentiation of perinatal hepatocytes by prematurely activating the genes with bivalent domains in hepatocytes. These bivalent domain-containing genes are involved in regulating hepatocyte maturation and liver fibrosis [67]. Therefore, EZH2 has critical roles in liver development, homeostasis, and regeneration (Figure 1).

## 4. EZH2 as a Therapeutic Target in NAFLD

EZH2 overexpression is frequently detected in HCC, and the mechanisms by which EZH2 promotes HCC are associated with tumor growth and metastasis [68,69]. Specifically, EZH2 silences tumor-suppressor microRNAs [70,71] and regulates the cell cycle, proliferation, and apoptosis [72]. 

Recently, the role of EZH2 in NAFLD progression has begun to be revealed. Mann et al. [73] have described liver fibrosis as being epigenetically regulated by methyl-CpG binding protein 2 (MeCP2) and EZH2. Transdifferentiation of HSCs into myofibroblasts is a key event in liver fibrosis, and this event is suppressed by peroxisome proliferator-activated receptor-gamma (PPARγ) [74]. During transdifferentiation, MeCP2 is recruited to the 5′ end of PPARγ and stimulates EZH2 expression. EZH2 methylates H3K27 to form a repressive chromatin structure in the 3′ exons of PPARγ resulting in down-regulation of PPARγ [73]. Moreover, EZH2 is up-regulated in the liver of CCl_4_-treated rats and promotes hepatic fibrosis by repressing the Wnt pathway antagonist Dkk-1 [75]. In addition, activation of the NAD-dependent deacetylase SIRT1 attenuates liver fibrosis through deacetylation of EZH2, which affects the stability of EZH2 and prevents myofibroblast generation [76]. 

Transforming growth factor β (TGF-β) is one of the most important cytokines expressed after liver damage [77]. TGF-β is increased in human hepatic fibrosis [78]. Up-regulation of TGF-β in activated HSCs occurs through multiple mechanisms [79]. Martin-Mateos et al. [80] demonstrated that TGF-β-mediated HSC activation depends on the level of EZH2. EZH2 suppression results in attenuation of TGF-β-induced activation of the major fibrogenic genes such as *FN1*, *COL1A1*, and *ASMA* in human primary HSCs [80]. Moreover, EZH2 overexpression promotes TGF-β-mediated HSC activation in vitro. Furthermore, administration of the EZH2 inhibitor GSK-503 attenuates liver fibrosis in CCl_4_-treated or bile duct ligation mouse models [80]. Therefore, EZH2 modulation could be a potential target for hepatic fibrosis treatment. By contrast, negative effects related to a decrease in EZH2 function in NAFLD have also been suggested. Vella et al. [81] showed that EZH2 is down-regulated both in livers from NAFLD rats and in HepG2 cells treated with free fatty acid. In these free fatty acid-treated HepG2 cells, treatment with 3-deazaneplanocin A (DZNep), an EZH2 inhibitor (see Section 5.1), induces lipid accumulation. These opposing effects might be related to differences in NAFLD models. In addition, DZNep indirectly inhibits various *S*-adenosyl-l-methionine (SAM)-dependent methyltransferases as well as EZH2, which also prevents a straightforward interpretation of these findings.

**Figure 2:**
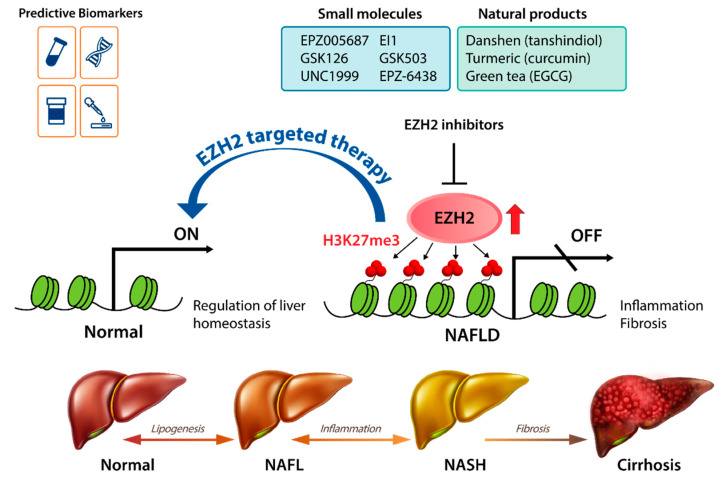
EZH2 as a therapeutic target in NAFLD. Abnormal activation of EZH2 may drive NAFLD progression, and EZH2-targeted therapy could be a promising strategy for NAFLD. A major class of small-molecule inhibitors of EZH2 is the SAM-competitive inhibitors, such as EPZ005687, EI1, GSK126, UNC1999, GSK503, and EPZ-6438. Natural products such as tanshindiols (the major active components of danshen), curcumin (a natural polyphenol from turmeric), and epigallocatechin-3-gallate (EGCG, the major chemical constituent of green tea) have EZH2-inhibiting activity. Development of predictive biomarkers to select patients suitable for EZH2-targeted therapy will improve the efficiency of this approach.

More recently, Lee et al. [82] showed that EZH2 has a critical role in liver inflammation and fibrosis using the STAM NASH mouse model. In the STAM NASH mouse model, streptozotocin is administered, which causes inflammation and insulin secretion impairment, resulting in a phenotype resembling advanced type 2 diabetes [83]. STAM NASH mice serve as a model of human NAFLD, from steatosis to fibrosis, with a phenotype similar to that of human clinical samples [84]. Treatment of STAM NASH mice with the EZH2 inhibitor EPZ-6438 or UNC1999 decreases the mRNA expression of inflammatory cytokines and fibrosis markers [82]. To sum up, overexpression of EZH2 may drive NAFLD progression, and pharmacologic inhibition of EZH2 could be a promising strategy for treating NAFLD (Figure 2).

## 5. EZH2 Inhibitors

### 5.1. Small-Molecule Inhibitors of EZH2

Many pharmaceutical companies have developed EZH2 inhibitors in light of evidence that EZH2 can be a driver of human diseases including cancer [22]. SAM is a universal methyl donor for catalytic reactions of histone methyltransferases. A major class of EZH2 inhibitors is the SAM-competitive inhibitors, such as EPZ005687, EI1, GSK126, UNC1999, GSK503, and EPZ-6438 (Table 1). 

DZNep is one of the purine nucleoside analogs, many of which are effective in the treatment of hematological malignancies and autoimmune disorders [85]. DZNep can inhibit EZH2 indirectly by interfering with SAM and SAH (*S*-adenosyl-l-homocysteine) metabolism, acting as an SAH hydrolase inhibitor [86]. Zeybel et al. [87] discovered that DZNep treatment inhibits liver fibrosis that results from multiple histone methylation modifications. They demonstrated that the in vivo modulation of HSC histone methylation is sufficient to suppress liver fibrosis by selectively targeting DZNep to HSC-derived myofibroblasts in the mouse [87]. This discovery represents an important proof of concept for epigenetic treatments targeting NAFLD. 

EPZ005687 is a selective inhibitor of EZH2 and displays >500-fold selectivity for EZH2 as compared with 15 other human protein methyltransferases and 50-fold selectivity over EZH1. It directly inhibits enzymatic activity of EZH2 rather than formation of PRC2 in an in vitro assay [88]. EI1 is another EZH2 inhibitor that directly binds to the enzyme and competes with the methyl group donor SAM. EI1-treated cells exhibit genome-wide loss of H3K27 methylation and activation of PRC2 target genes [89]. 

The selectivity of GSK126 for EZH2 is >1000-fold higher than its selectivity for 20 other human methyltransferases, and it is 150-fold more selective for EZH2 than for EZH1 [90]. GSK126 ameliorates disease severity in liver failure mice and down-regulates circulating and hepatic proinflammatory cytokines, especially TNF, by reducing H3K27me3 [91]. UNC1999, an analog of GSK126, shows a 10-fold selectivity for EZH2/EZH1. UNC1999 is highly selective for EZH2 and EZH1 over a broad range of epigenetic and non-epigenetic targets [92]. 

EPZ-6438 (Tazemetostat) shows a 35-fold selectivity versus EZH1 and >4500-fold selectivity relative to 14 other histone methyltransferases [93]. EPZ-6438 was developed from EPZ005687 with improved pharmacokinetic properties including good oral bioavailability [93,94]. EPZ-6438 received approval in January 2020 in the USA for the treatment of epithelioid sarcoma. The recommended dosage regimen is 800 mg twice daily, administered orally with or without food, until disease progression or unacceptable toxicity occurs. EPZ-6438 is also undergoing clinical development in various countries worldwide for use in several other tumor types, including diffuse large B-cell lymphoma and mesothelioma [95]. 

### 5.2. Natural Products with EZH2-Inhibiting Activity

With respect to safe alternative approaches for treating NAFLD, we are also interested in natural products that have EZH2-inhibiting activity. Shahabipour et al. [98] summarized various natural products that suppress EZH2. 

The root of danshen (*Salvia miltiorrhiza*) has significant pharmacological activities with respect to a variety of human diseases. Clinical trials using danshen for the treatment of liver cirrhosis have suggested that danshen may promote the curative efficacy of treatments for liver cirrhosis [99]. Woo et al. [100] found that tanshindiols, the major active components of danshen, function as a SAM-competitive inhibitor and can inhibit EZH2 histone methyltransferase activity. 

Turmeric (rhizomes of *Curcuma longa*) has been widely used not only as a culinary spice but also as a medicinal agent in Eastern medicine [101]. Curcumin, a natural polyphenol from turmeric, has been widely studied for its anti-inflammatory, anti-angiogenic, anti-oxidant, wound healing, and anti-cancer effects [102]. A clinical trial of curcumin for patients with NAFLD showed a significant reduction in liver fat content; body mass index; and serum levels of total cholesterol, low-density lipoprotein cholesterol, triglycerides, aspartate aminotransferase, alanine aminotransferase, glucose, and glycated hemoglobin as compared with the placebo group [103]. Moreover, curcumin was safe and well tolerated during the course of the trial [103]. Curcumin was found to suppress EZH2 expression through the MAPK pathway in breast cancer cells [104] and through let-7c and miR-101 in lung cancer cells [105].

Green tea (*Camellia sinensis*) is popular worldwide. Mansour-Ghanaei et al. [106] found significant effects of green tea supplementation on NAFLD treatment through the meta-analysis of clinical trials. Epigallocatechin-3-gallate (EGCG), the major chemical constituent of green tea, suppresses EZH2 expression in skin cancer cells [107] and causes anti-cancerous epigenetic changes in acute promyelocytic leukemia cells [108]. In summary, the properties of these natural compounds that relate to NAFLD treatment might be at least in part mediated by EZH2-suppressing activity. These natural products have the advantages of being safe and of having multi-target action, such that they could be used as nutraceuticals in combination with other NAFLD agents that do not have overlapping toxicity [98].

## 6. Conclusions

EZH2 has a key role in liver development and homeostasis, and abnormal activation of EZH2 can lead to NAFLD progression (Figure 2). Recent in vitro and in vivo studies have shown the therapeutic effects of EZH2 inhibitors in NAFLD, but our understanding of the molecular mechanisms involved is still limited. Further research is necessary to prove the direct relationship between EZH2 inhibition and NAFLD improvement. More accurate models that better mimic the NAFLD disease spectrum will provide increased mechanistic understanding [109]. Recent human liver organoid models made up of multiple cell types may facilitate the discovery of effective new drugs for NAFLD therapy [110,111]. Determining the detailed actions of EZH2 in the pathogenesis and progression of NAFLD should lead to new strategies to reverse this disease.

Several small molecules have been developed as EZH2 inhibitors. Encouragingly, EPZ-6438 was clinically approved for the treatment of epithelioid sarcoma and may be approved for the treatment of other diseases in the future. Peptides such as SAH-EZH2 (stabilized alpha-helix of EZH2) [112] and antisense oligonucleotides [113] can also inhibit EZH2. In addition, some natural products show EZH2-inhibiting effects. 

To develop an EZH2 inhibitor as a drug for NAFLD treatment, low toxicity and high efficacy are required. Combination therapy with other treatments such as chemotherapy, immunotherapy, or microbiome therapy could have a synergistic effect if there are no overlapping toxicities. Importantly, the development of predictive biomarkers to select patients suitable for EZH2-targeted therapy will improve the efficiency of this approach [114]. Moreover, non-invasive biomarkers are urgently needed in NAFLD therapy. Recent genomic and epigenomic studies of liver tissue samples, blood, and feces from NAFLD patients should facilitate the development of future NAFLD treatments. 

## Figures and Tables

**Table 1:** Small molecules with EZH2-inhibiting activity.

Compound	Mechanism	Selectivity *	Ref(s).
DZNep	SAH hydrolase inhibitor	Unknown	[86,96]
EPZ005687	SAM-competitive inhibitor of PRC2	>500-fold over other HMTs, ~50-fold over EZH1	[88]
EI1	SAM-competitive inhibitor of PRC2	>10,000-fold over other HMTs, ~90-fold over EZH1	[89]
GSK126	SAM-competitive inhibitor of PRC2	>1000-fold over other HMTs, 150-fold over EZH1	[90]
UNC1999	SAM-competitive inhibitor of PRC2	10,000-fold over other HMTs, 10-fold over EZH1	[92]
GSK503	SAM-competitive inhibitor of PRC2	>4000-fold over other HMTs, 200-fold over EZH1	[97]
EPZ-6438	SAM-competitive inhibitor of PRC2	>4500-fold over other HMTs, 35-fold over EZH1	[93,94]

* Selectivity data are presented relative to inhibition of other HMTs (histone methyl transferases) and EZH1.

## Abbreviations

BET	Bromodomain and extra-terminal domain
CBX	Chromobox domain
ChREBP	Carbohydrate response element binding protein
DNMT	DNA methyltransferase
DZNep	3-deazaneplanocin A
EED	Embryonic ectoderm development
EGCG	Epigallocatechin-3-gallate
EZH2	Enhancer of zeste homolog 2
H3K27me3	Trimethylation on Lys 27 of histone H3
H3K4me3	Trimethylation on Lys 27 of histone H3
H3K9me2	Dimethylation on Lys 9 of H3
HAT	Histone acetyltransferase
HCC	Hepatocellular carcinoma
HDAC	Histone deacetylase
HMT	Histone methyl transferase
HPH	Human polyhomeotic homolog
HSC	Hepatic stellate cell
KDM	Histone lysine demethylase
KMT	Histone lysine methyltransferase
MeCP2	Methyl-CpG binding protein 2
NASH	Non-alcoholic steatohepatitis
PPARγ	Peroxisome proliferator-activated receptor-gamma
PcG	Polycomb group
PCGF	Polycomb group ring finger protein
Phf2	Plant homeodomain finger 2
PRC	Polycomb repressive complex
SAH	*S*-adenosyl-l-homocysteine
SAM	*S*-adenosyl-l-methionine
SUZ12	Suppressor of zeste 12
